# A Multiplex Asymmetric Reverse Transcription-PCR Assay Combined With an Electrochemical DNA Sensor for Simultaneously Detecting and Subtyping Influenza A Viruses

**DOI:** 10.3389/fmicb.2018.01405

**Published:** 2018-06-27

**Authors:** Lili Xu, Xiwen Jiang, Yun Zhu, Yali Duan, Taosheng Huang, Zhiwen Huang, Chunyan Liu, Baoping Xu, Zhengde Xie

**Affiliations:** ^1^Beijing Key Laboratory of Pediatric Respiratory Infection Diseases, Key Laboratory of Major Diseases in Children, Ministry of Education, National Clinical Research Center for Respiratory Diseases, National Key Discipline of Pediatrics (Capital Medical University), Beijing Pediatric Research Institute, Beijing Children’s Hospital, Capital Medical University, National Center for Children’s Health, Beijing, China; ^2^DAAN Gene Co., Ltd., Sun Yat-sen University, Guangzhou, China; ^3^The Medicine and Biological Engineering Technology Research Center of the Ministry of Health, Guangzhou, China; ^4^National Clinical Research Center for Respiratory Diseases, Department of Respiratory, Beijing Children’s Hospital, Capital Medical University, National Center for Children’s Health, Beijing, China

**Keywords:** multiplex asymmetric reverse transcription-PCR assay, electrochemical DNA sensor, Luminex RVP assay, influenza A viruses, subtype

## Abstract

The reliable and rapid detection of viral pathogens that cause respiratory infections provide physicians several advantages in treating patients and managing outbreaks. The Luminex respiratory virus panel (RVP) assay has been shown to be comparable to or superior to culture/direct fluorescent-antibody assays (DFAs) and nucleic acid tests that are used to diagnose respiratory viral infections. We developed a multiplex asymmetric reverse transcription (RT)-PCR assay that can simultaneously differentiate all influenza A virus epidemic subtypes. The amplified products were hybridized with an electrochemical DNA sensor, and the results were automatically acquired. The limits of detection (LoDs) of both the Luminex RVP assay and the multiplex RT-PCR-electrochemical DNA sensor were 10^1^ TCID_50_ for H1N1 virus and 10^2^ TCID_50_ for H3N2 virus. The specificity assessment of the multiplex RT-PCR-electrochemical DNA sensor showed no cross-reactivity among different influenza A subtypes or with other non-influenza respiratory viruses. In total, 3098 respiratory tract specimens collected from padiatric patients diagnosed with pneumonia were tested. More than half (43, 53.75%) of the specimens positive for influenza A viruses could not be further subtyped using the Luminex RVP assay. Among the remaining 15 specimens that were not subtyped, not degraded, and in sufficient amounts for the multiplex RT-PCR-electrochemical DNA sensor test, all (100%) were H3N2 positive. Therefore, the sensitivity of the Luminex RVP assay for influenza A virus was 46.25%, whereas the sensitivity of the multiplex RT-PCR-electrochemical DNA sensor for the clinical H1N1 and H3N2 specimens was 100%. The sensitivities of the multiplex RT-PCR-electrochemical DNA sensor for the avian H5N1, H5N6, H9N2, and H10N8 viruses were 100%, whereas that for H7N9 virus was 85.19%. We conclude that the multiplex RT-PCR-electrochemical DNA sensor is a reliable method for the rapid and accurate detection of highly variable influenza A viruses in respiratory infections with greater detection sensitivity than that of the Luminex xTAG assay. The high mutation rate of influenza A viruses, particularly H3N2 during the 2014 to 2016 epidemic seasons, has a strong impact on diagnosis. A study involving more positive specimens from all influenza A virus epidemic subtypes is required to fully assess the performance of the assay.

## Introduction

Influenza A virus causes morbidity and mortality in humans, and this is overstated during epidemic years, which is a major public health concern (Neuzil et al., 2000; Petrova and Russell, 2018). Based on the antigenic specificity of haemagglutinin (HA) and neuraminidase (NA) proteins, influenza A viruses can be classified into subtypes H1–H18 and N1–N11, respectively (Wu et al., 2014; Pinsent et al., 2016). Currently, H1N1 and H3N2 are the most prevalent human subtypes and continue to co-circulate as two subtypes. Outbreaks of avian influenza viruses, such as H5N1, H5N6, H7N9, H9N2, and H10N8, have shown that these viruses can bypass the species barrier from poultry to humans, causing mortality in both species (Li and Cao, 2017). Rapid detection and subtyping are the first steps in the characterization of influenza A viruses, and can facilitate the appropriate treatments to improve patient clinical outcomes and significantly reduce hospital costs (Bonner et al., 2003).

Traditionally, respiratory viral infections have been diagnosed by culture, a rapid antigen test, or a direct fluorescent-antibody assay (DFA) (Uyeki, 2003). However, many studies have demonstrated that molecular diagnostic assays have superior sensitivity over conventional assays, and these assays are currently considered the new gold standard (Weinberg et al., 2004; Kuypers et al., 2006; Letant et al., 2007; Hodinka and Kaiser, 2013). The introduction of multiplex PCR assays has increased the efficiency of the routine molecular diagnosis of various viruses and has been shown to be cost-effective (Auburn et al., 2011; Jansen et al., 2011; Choudhary et al., 2013). The RVP assay (Luminex Molecular Diagnostics Inc., Toronto, Canada) is a CE-marked, commercially available kit based on suspension microarray technology that enables the detection of many viruses in a single reaction (Krunic et al., 2007; Merante et al., 2007). The Luminex RVP assay has been shown to be more sensitive and specific than culture and antigen detection (Wong et al., 2009; Gadsby et al., 2010). However, the cost of the kit and instrument is a major disadvantage of the Luminex RVP assay.

Biosensors represent a newly developing detection technique that combines biochemical, electrochemical, medical and electronic techniques. Recently, electrochemical DNA sensors have received much interest in many fields due to their unique advantages, such as their innately high sensitivity, simple instrumentation, automation and low cost in the detection of infectious diseases, clinical diagnosis of pathogens, genetic diagnoses, environmental pollutant determination, food safety determination, epidemiological studies, and forensic identification (Kerman et al., 2004). A gold electrode in an electrochemical DNA sensor is functionalized using a DNA probe sequence, oligo phenylmethyl molecular wires, and polyethylene glycol insulator molecules. The target is captured on the electrode and hybridizes to a second reporter sequence labeled with ferrocene designated the signal probe. The electrochemical detection of hybridization is primarily based on the differences in the electrochemical conduction of the labels with or without double-stranded DNA (dsDNA) or single-stranded DNA (ssDNA). Additionally, the use of a sandwich structure for DNA hybridization further improves the selective recognition of the DNA electrochemical sensor; thus, this DNA electrochemical sensor has a high specificity (Kerman et al., 2004).

In this study, we developed and evaluated the diagnostic performance of a multiplex asymmetric RT-PCR-electrochemical DNA sensor for the simultaneous differentiation of all influenza A virus epidemic subtypes. The Luminex RVP assay and commercial real-time PCR kits were used to separately detect human and avian influenza viruses for comparison.

## Materials and Methods

### Positive Control Viruses

The influenza virus isolates A/California/07/2009 (H1N1), A/duck/Wuxi/2/2013 (H2N1), A/Brisbane/10/2007 (H3N2), A/Anhui/1/2005 (H5N1), A/Guangzhou/39715/2014 (H5N6), A/Anhui/1/2013 (H7N9), A/duck/Zhejiang/3/2002 (H9N2), and A/duck/2/Hebei/2014 (H10N8) were kindly provided by Prof. Sanhong Yin and Dr. Xianpeng Zhang from the Dongguan Animal Disease Prevention and Control Center. All experiments involving virus cultures of highly pathogenic viruses were conducted under BSL-3 containment.

### Clinical Sample Collection

Between November 2014 and April 2016, 3098 pediatric pneumonia patients with a median age of 2.17 years old (ranging from 7 days old to 16.08 years old) were enrolled at the Pathogen Surveillance Network of Beijing Children’s Hospital. Nasopharyngeal aspirate or throat swab specimens were collected from each patient. This study was performed in strict accordance with human subject protection guidance and was approved by the Ethical Review Committee of Beijing Children’s Hospital. Written informed consent was obtained on the participants’ behalf from their parents or guardians.

### Primer and Probe Design

The nucleotide sequences of the influenza A virus matrix (M) gene; the H1, H2, H3, H5, H7, H9, and H10 genes; and the N1, N2, N6, N8, and N9 genes were retrieved from the GenBank database and aligned using the BioEdit v7.1.3 software. The primers were designed and analyzed using the Oligo 7.0 software and aligned using BLAST with relative sequences in GenBank to verify the specific amplification. The top-rated primers and probes from the Oligo 7.0 software with non-annealing, irrelevant sequences in GenBank evaluated by using BLAST were chosen for use. Specific forward and reverse primers were used for M gene amplification, whereas specific forward primers and universal reverse primers were used for HA and NA gene amplification. The capture probes were C6 S-S-labeled (Thiol Modifier), and the signal probes were labeled with ferrocene. The finalized primers and probes are listed in **Table 1**.

**Table 1:** The primers and probes used for the multiplex asymmetric RT-PCR assay in this study.

Primer	Sequence (5′–3′)	Location	Tm (°C)	Probe	Sequence (5′–3′)	Location	Reference strain GenBank Number
M-forward	TCTTCTAACCGAGGTCGAAACG	31–52	62.7	M-CP	TTGTATTCACGCACACCGTGC - C6 S-S	210–230	HM142730.1
M- reverse	CGTCTACGCTGCAGTCCT	241–258	57.9	M-SP	Fc - CCCAGTGAGCGAGG	230–243	
H1- forward	TAATAGCACCATGGTATGCTTT	785–806	54.7	H1-CP	TCATTGAAGGGGGATGGACAGGA - C6 S-S	1049–1071	FN436023.1
				H1-SP	Fc - ATGATAGATGGGTGGTATGG	1072–1091	
H2- forward	ATAGAAGGRGGATGGCAAGG	1057–1076	56.0	H2-CP	GCAGCAGACAAAGAGTCCA- C6 S-S	1166–1184	KP767619.1
				H2-SP	Fc - CTCAAAAGGCAATTGATGGAATCACCAAC	1185–1213	
H3- forward	TTCACTTGGACAGGAGTAAC	421–440	51.9	H3-CP	ATGCCAAACAATGACAATTTTGAC - C6 S-S	550–573	KY415635.1
				H3-SP	Fc - AAACTATACATCTGGGG	574–590	
H5- forward	ATCATTGACAAAATGAACACTCAR	1201–1225	57.6	H5-CP	ATGGARGACGGATTCCTAGATGTCTGG - C6 S-S	1288–1314	DQ095621.2
				H5-SP	Fc - ACTTATAAYGCTGAACT	1315–1331	
H7- forward	TAAGCAGCGGCTACAAAG	1550–1567	56.3	H7-CP	TGGTTTAGCTTCGGGGCATCAT - C6 S-S	1579–1600	KP765952.1
				H7-SP	Fc - GTTTTCTTCTTCTAGCC	1601–1617	
H9- forward	TCAACAAACTCCACAGAAAC	79–98	49.6	H9-CP	CTAACAGAAAACAATGTYCCT - C6 S-S	109–129	KT699056.1
				H9-SP	Fc - GTGACACATGCCAAAGAA	130–147	
H10- forward	ATAGCACCGAGCCGAGTTAG	803–822	60.9	H10-CP	CACCAATAGACAATAATTGTGAGTCCA - C6 S-S	861–887	KY402019.1
				H10-SP	Fc - AATGTTTTTGGAGAGGG	888–904	
N1- forward	AGTGCTCCTGTTATCCTGATG	773–793	51.7	N1-CP	TATATATGCAGTGGAGTTTTCGGAGA - C6 S-S	886–911	KF667692.1
				N1-SP	Fc - CAATCCACGCCCCAATG	912–928	
N2- forward	AAGGACAACTCAATTAGGCTYTC	314–336	56.2	N2-CP	ACCAAACAAGTGTGCATAGCAT - C6 S-S	521–542	KP767935.1
				N2-SP	Fc - GGTCTAGTTCAAGCTG	543–558	
N6- forward	TGCATAGGATGGTCAAGCA	526–544	49.0	N6-CP	AGGATGTCAATATGCATATCAGGA - C6 S-S	568–591	KM251485.1
				N6-SP	Fc - CCGAATAACAATGCATC	592–608	
N8- forward	CAATGAAACAGTAAGGGTTGAGA	159–181	55.0	N8-CP	ATTGTGTGATGCTAAGGGTTTCG - C6 S-S	264–286	KY402021.1
				N8-SP	Fc - CACCCTTTTCCAAAGAC	287–303	
N9- forward	ACAACCAACACAAGCCAAAC	148–167	57.3	N9-CP	TTTCAATAACTTAACTAAAGGGCTC - C6 S-S	237–261	KP416240.1
				N9-SP	Fc - TGTACTATAAATTCATGGC	262–280	
Universal reverse 01	ATATCGTCTCGTATTAAGTAGAAACAAG	1716–1743	60.6	/	/	/	FN436023.1
Universal reverse 02	ATATGGTCTCGTATTAGTAGAAACAAGGAG	1357–1386	61.6	/	/	/	KF667692.1
RNaseP - forward	AGATTTGGACCTGCGAGCG	/	55.0	RNaseP - probe	FAM - TTCTGACCTGAAGGCTCTGCGCG - BHQ-1	/	/
RNaseP - reverse	GAGCGGCTGTCTCCACAAGT		59.0				
18S rRNA forward	AGCGAAAGCATTTGCCAAGA	/	53.0	/	/	/	/
18S rRNA reverse	AGTCTCGTTCGTTATCGGAATT		57.0				

*CP, capture probe; SP, signal probe; and Fc, ferrocene.*

### Multiplex Reverse Transcription-PCR and DNA Sensor Hybridization

Total nucleic acids were extracted from the clinical samples using a NucliSens easyMAG system (BioMérieux, Marcy-l’Etoile, France) according to the manufacturer’s instructions. A mixed positive control containing each positive control influenza virus and one negative control consisting of cultured, uninfected human epithelial cells was processed with each batch of clinical specimens.

For each sample, reverse transcription-PCR was performed using three microcentrifuge tubes with three different PCR mixes. Tube A targeted H1, H2, H3, H5, H7, H9, and H10 genes of influenza A viruses; Tube B targeted N1, N2, N6, N8, and N9 genes of influenza A viruses; and Tube C targeted the M gene of influenza A virus. The 50-μL reaction mixture consisted of 10 mM of Tris-HCl (pH 8.3), 3.5 mM of MgCl_2_, 20 mM of KCl, 200 mM of dNTPs, forward primers (10 μM each), reverse primers (1 μM each) and 50 ng of extracted RNA. PCR was performed using an ABI9700 Thermal Cycler (Applied Biosystems, United States). The PCR conditions were as follows: (Neuzil et al., 2000) 50°C for 30 min; (Petrova and Russell, 2018) 95°C for 15 min; (Pinsent et al., 2016) 45 cycles of 94°C for 30 s, 50°C for 30 s, and 72°C for 30 s; and (Wu et al., 2014) a final extension at 72°C for 7 min. Each batch of PCR runs included nucleic acid extracted from the positive mix and negative controls, and a further amplification and quantification of the RNaseP gene (forward, 5′-AGATTTGGACCTGCGAGCG-3′; reverse, 5′-GAGCGGCTGTCTCCACAAGT-3′; and probe, 5′-TTCTGACCTGAAGGCTCTGCGCG-3′) was carried out as an internal reference control (World Health Organisation [WHO], 2013).

Because unequal amounts of reverse primers were used in the asymmetric PCR process, after PCR amplification, large amounts of ssDNA that could be directly hybridized were generated using the signal probes (100 μM each), 10 μL of foetal bovine serum (FBS), and 20 μL of sodium perchlorate for 30 min without heat denaturation.

The electrochemical DNA sensor consisted of 72 gold electrodes. Electrode pretreatment and capture probe immobilization were performed according to a previously reported protocol (Lao et al., 2005). The electrochemical signal was recorded when the target DNA hybridized to the capture probe and ferrocene-labeled signal probe, thereby connecting the reporter molecule ferrocene to the self-assembled monolayer on the gold electrode. Voltmeter measurements and data analysis were performed using a DA9100 Electrochemical Workstation (DAAN Gene Co., Ltd.). All positive amplicons were further confirmed by sequencing.

### Luminex RVP Assay

A Luminex RVP assay was performed to detect 18 common respiratory viral pathogens and subtypes, including influenza A, influenza A subtype H1, influenza A subtype H3, 2009 H1N1, influenza B, HAdV, HPIV 1–4, RSV A and B, human metapneumovirus (HMPV), enteroviruses and rhinoviruses (EV/Rh), human coronavirus (HCoV) HKU1, 229E, NL63, and OC43, and human bocavirus (HBoV), in the nucleic acid samples. An internal positive control was added to each specimen before the nucleic acid extraction procedure was performed, and a positive PCR control (Lambda DNA) was added to each PCR run according to the manufacturer’s manual. In this study, blank VTM served as a negative control for nucleic acid extraction.

### Comparison of the Sensitivity and Limits of Detection (LoDs) Between the Luminex RVP Assay and Multiplex RT-PCR-Electrochemical DNA Sensor

To compare the detection sensitivities of the two methods, a true positive was defined as a positive specimen in both tests or any specimen positive in only one test that was further confirmed by sequencing. All specimens with concordant or discordant results were tested using both methods in duplicate.

We compared the LoDs of these two detection methods using 10-fold serial dilutions of positive control viruses ranging from 10^4^ TCID_50_ to 10^0^ TCID_50_. The LoD procedure was carried out as described by Bustin et al. (2009). All dilutions were detected in three duplex reactions.

### Specificity Detection of the Multiplex RT-PCR-Electrochemical DNA Sensor

The specificity of the multiplex RT-PCR assay was evaluated by detecting pediatric pneumonia specimens that were positive for other pathogens, including RSV, HAdV, HMPV, HPIV, EV/Rh, HCoV, and HBoV (five positive specimens for each pathogen) using a Luminex RVP assay.

### Comparison of Detection Sensitivity for Avian Influenza Virus Positive Specimens Between the Multiplex RT-PCR-Electrochemical DNA Sensor and Real-Time PCR Kits

In total, 186 specimens positive for avian influenza viruses collected from poultry or birds in southern China from 2006 to 2014 and verified by gene sequencing were used to assess the detection sensitivity of the multiplex RT-PCR-electrochemical DNA sensor for avian influenza viruses. Several commercial real-time PCR kits produced by DAAN Gene Co., Ltd. were used to detect the H5N1, H5N6, H7N9, H9N2, and H10N8 viruses for comparison. Sample quality was controlled by using 18S rRNA as housekeeping control gene with bird-conserved primers (forward, 5′-AGCGAAAGCATTTGCCAAGA-3′; reverse, 5′-AGTCTCGTTCGTTATCGGAATT-3′), validated against a variety of bird species (including chicken, duck, goose, and crow) and tissues (spleen, brain, lung, trachea, cecum, and liver) (Kerman et al., 2004). All avian specimens were kindly provided by Prof. Sanhong Yin and Dr. Xianpeng Zhang at the Dongguan Animal Disease Prevention and Control Center, and the study was conducted under BSL-3 containment. The animal specimen usage was approved by the Ethical Review Committee of Dongguan Animal Disease Prevention and Control Center.

### Statistical Analysis

The exact (Clopper-Pearson) method was used to calculate the 95% confidence intervals (95% CI). The sensitivity estimates for each virus were calculated based on two-by-two tables from the entire prospective data set.

## Results

We first assessed the LoD and specificity of the developed multiplex RT-PCR-electrochemical DNA sensor. The LoD of the Luminex RVP assay and multiplex RT-PCR-electrochemical DNA sensor was evaluated using serially diluted positive control viruses for H1N1 or H3N2. Based on three duplex reactions, an excellent reproducibility result showed that the LoDs using both methods were 10^1^ TCID_50_ for H1N1 and 10^2^ TCID_50_ for H3N2. According to the specificity analysis, the multiplex RT-PCR assay developed in this study specifically amplified 7 HA genes, 5 NA genes, and the M gene from the influenza A viruses. No cross-reactivity was observed among the subtypes, and no spurious PCR amplification or voltmeter measurements were observed in the specimens positive for RSV, HAdV, HMPV, HPIV, EV/Rh, HCoV, and HBoV.

Second, the clinical specimens were used to compare the sensitivity of the multiplex RT-PCR-electrochemical DNA sensor using comparative methods. Using the Luminex RVP assay, 149 (4.81%) influenza-positive cases were detected among 3098 pediatric pneumonia patients between November 2014 and April 2016, including 79 (2.55%) cases infected with influenza A, 71 (2.29%) cases infected with influenza B, and 1 case co-infected with influenza A and B. The influenza A positive specimens were simultaneously differentiated into H1N1 and H3N2 using the Luminex RVP assay; however, only 23.75% (19/80) and 22.50% (18/80) of the specimens were identified as 2009 H1N1 and H3N2, respectively, and 43 specimens (53.75%) could not be subtyped using the Luminex RVP assay.

The influenza A-positive specimens which were verified by the Luminex RVP assay were then tested using the multiplex RT-PCR-electrochemical DNA sensor. For all 19 2009 H1N1-positive and 18 H3N2-positive specimens detected by the Luminex RVP assay, the results obtained using the multiplex RT-PCR-electrochemical DNA sensor were in total agreement. However, for the 42 non-subtyped influenza A specimens detected by the Luminex RVP assay, due to repeated detection based on the negative results, only 17 specimens were available in sufficient amounts for the multiplex RT-PCR-electrochemical DNA sensor test. Two of these specimens were negative for the RNaseP gene, which indicated that these two specimens were too degraded for detection. The remaining 15 (88.24%) specimens were H3N2-positive, and no H1N1-positive specimens were detected. All the positive amplicons detected by the multiplex RT-PCR-electrochemical DNA sensor were confirmed by sequencing. To further detect the sensitivity of the multiplex RT-PCR-electrochemical DNA sensor, we randomly selected 96 specimens that showed negative results for all viruses by using the Luminex RVP assay. In accordance with the Luminex RVP assay, no influenza virus was detected.

In summary, based on the above data, the sensitivity of the Luminex RVP assay for the influenza A virus was 46.25% (95% CI, 45.03–47.47%). The true number of positive specimens using the multiplex RT-PCR-electrochemical DNA sensor was 52 (37 positive by both tests and 15 specimens positive by the multiplex RT-PCR-electrochemical DNA sensor, followed by further confirmation by sequencing). The sensitivity of the multiplex RT-PCR-electrochemical DNA sensor for the influenza A virus (including H1N1 and H3N2) was 100% (95% CI, 93.15–100%) (**Table 2**).

**Table 2:** Comparison of the detection sensitivity for the clinical H1N1- and H3N2-positive specimens between the Luminex RVP assay and the multiplex RT-PCR-electrochemical DNA sensor.

Virus	Luminex RVP assay			Multiplex RT-PCR-electrochemical DNA sensor		
	Number of true positive specimens	Number with positive results	Sensitivity %) (95% CI)	Number of true positive specimens	Number with positive results	Sensitivity % (95% CI)
Influenza A viruses	80	37	46.25% (45.03–47.47)	52	52	100% (93.15–100)
H1N1	ND	19	/	19	19	100% (82.35–100)
H3N2	ND	18	/	33	33	100% (89.42–100)

Furthermore, to evaluate the practical application of the multiplex RT-PCR-electrochemical DNA sensor for the detection of avian influenza viruses, which were lacking in the pediatric pneumonia specimens, we compared the sensitivity of this detection method using a series of real-time PCR kits to separately detect the H5N1, H5N6, H7N9, H9N2, and H10N8 viruses. According to the real-time PCR results, of the 186 avian specimens, 18S rRNA genes showed equal orders of magnitude and 16, 56, 27, 82, and 5 specimens were positive for H5N1, H5N6, H7N9, H9N2, and H10N8, respectively. The detection sensitivities of these real-time PCR kits were 100% (186/186; 95% CI, 98.04–100%). Using the multiplex RT-PCR-electrochemical DNA sensor, the detection sensitivities for H5N1, H5N6, H9N2, and H10N8 were also 100%. However, 4 H7N9-positive specimens showed negative results, indicating that the detection sensitivity for the avian H7N9 virus was 85.19% (23/27; 95% CI, 82.61–87.76%), while that for all detected avian influenza viruses was 97.85% (182/186; 95% CI, 97.70–98.00%) (**Table 3**). Compared to the real-time PCR results, the copy numbers of the 4 negative specimens detected using the multiplex RT-PCR-electrochemical DNA sensor were less than 10^2^ copies/mL, and the other 23 specimens with positive results showed viral copy numbers greater than 10^2^ copies/mL. Thus, specimens with less than 10^2^ copies/mL H7N9 viral DNA may not be detected by using the multiplex RT-PCR-electrochemical DNA sensor. We also randomly selected 50 avian specimens that showed negative results by using real-time PCR for all avian influenza viruses, aiming to detect influenza viruses with the multiplex RT-PCR-electrochemical DNA sensor; however, no positive samples were found.

**Table 3:** Comparison of the detection sensitivity for the avian influenza virus-positive specimens between the multiplex RT-PCR-electrochemical DNA sensor and the real-time PCR kits.

Virus	Number of true positive specimens	Multiplex RT-PCR-electrochemical DNA sensor		Real-time PCR	
		Number with positive results	Sensitivity % (95% CI)	Number with positive results	Sensitivity % (95% CI)
H5N1	16	16	100% (79.41–100)	16	100% (79.41–100)
H5N6	56	56	100% (93.62–100)	56	100% (93.62–100)
H7N9	27	23	85.19% (82.61–87.76)	27	100% (87.23–100)
H9N2	82	82	100% (95.60–100)	82	100% (95.60–100)
H10N8	5	5	100% (47.82–100)	5	100% (47.82–100)
Total	186	182	97.85% (97.70–98.00)	186	100% (98.04–100)

## Discussion

Nucleic acid detection, particularly PCR-based approaches used to detect the influenza A virus, has recently expanded exponentially. Several multiplex RT-PCR methods have been developed for the identification of influenza A viruses (Chang et al., 2008; Chen et al., 2011; Fang et al., 2011; Tang et al., 2012; Wu et al., 2013). Electrochemical DNA sensors represent a novel developing technique that combines nucleic acid hybridization with electronic techniques and is simple, reliable, inexpensive, sensitive and selective for genetic detection; this method has become a hot topic in the fields of biochemistry and medicine. In this study, we developed a multiplex asymmetric PCR assay to simultaneously differentiate seven HA (H1, H2, H3, H5, H7, H9, and H10) and five NA (N1, N2, N6, N8, and N9) subtypes from three reaction mixtures and achieved detection using an electrochemical DNA sensor in less than 30 min. Multiplexing technologies are becoming increasingly popular since they offer increased testing throughput capacity and can reduce overall cost. The frequently reduced sensitivity and specificity are disadvantages of multiplex molecular assays. In particular, the hybridization of a labeled target DNA can be less than optimal due to the steric hindrance of the microarray surfaces, leading to the preferential binding of the target to the non-labeled antisense strand (Shchepinov et al., 1997). In this study, the primers and probes were designed and homologically aligned using BLAST. All the relative sequences in GenBank were used to verify the specific amplification rather than by downloading limited gene sequences to identify conserved regions that may be unsuitable for all strains collected at various times and locations worldwide. We used this multiplex RT-PCR-electrochemical DNA sensor to test clinical specimens from the 2017 autumn–winter season. Based on our results, the application of this method exhibited perfect performance in terms of the sensitivity and specificity in detecting clinical influenza A viruses (unpublished data, not shown).

Furthermore, the sensitivity of the assay was greatly improved by adapting an asymmetric PCR reaction based on temperature-differential primer design. Asymmetric PCR could result in a higher sensitivity than symmetric PCR because the target strand alone is a higher proportion of the product, which can non-competitively hybridize with the probe. Asymmetric PCR with a high sensitivity and specificity has been developed for the detection of various pathogens, such as influenza B virus, adenovirus, HIV, *Treponema pallidum*, *Ureaplasma urealyticum*, and *Chlamydia trachomatis* (Poddar, 2000; Cao et al., 2006; Tang et al., 2009; Wong et al., 2014).

The Luminex RVP is a multiplex PCR assay approved by the United States Food and Drug Administration (FDA). This assay has been shown to be comparable or superior to culture/DFA and nucleic acid tests in the diagnosis of respiratory viral infections (Pabbaraju et al., 2008; Jokela et al., 2012). However, in this study, the sensitivity of the Luminex RVP assay for influenza A viruses during the 2014–2016 seasons was only 46.25%. The sensitivity of the Luminex RVP assay for influenza A virus was 54.2% in a report by Choudhary et al. (2016), 68.8% in a report by Gadsby et al. (2010), and only 14% for H1N1 and 0% for H3N2 in a report by Raymaekers et al. (2011). In contrast, the sensitivity of the Luminex RVP assay for the influenza A virus was reported to be 98.0% by Pabbaraju et al. (2008), 96.6% by Kim et al. (2013), and 100% for H1N1 and 92.9% for H3N2 by Popowitch et al. (2013). Because the LoD of both methods was highly consistent, we hypothesize that the sensitivity results were inconsistent because the specimens were collected during different epidemic seasons from different regions worldwide, and the sequence variation rate of the influenza A viruses in the primer-probe binding region of the Luminex RVP assay is high, leading to negative results. Munro described the development of a multiplex RT-PCR assay using Luminex microarray hybridization to detect influenza virus subtypes and showed sensitivities of 97.3% for FluA, 90.5% for H1, 96.9% for H3, and 88.9% for FluB in 2010–2011 season specimens, whereas the detection of the 2011–2012 season specimens showed a sensitivity of 100% for FluA, 89.9% for H1, 96.4% for H3, and 95.6% for FluB (Munro et al., 2013). This study supported our hypothesis that the high mutation rate of influenza A viruses in various epidemic seasons has a strong impact on the sensitivity of the Luminex RVP assay for influenza A virus detection due to primer/probe mismatches. In this study, of the remaining 15 specimens that were non-subtyped by the Luminex RVP assay, not degraded, and present in sufficient amounts for the multiplex RT-PCR-electrochemical DNA sensor test, all (100%) were H3N2-positive. Thus, the mutation rate of the H3N2 viruses during the 2014–2016 epidemic seasons was much higher than that of the H1N1 viruses in China. Genetic surveillance of the H3N2 viruses from 2014 to 2016 indeed showed multiple substitutions in the antigenic sites and reassortant events worldwide (Skowronski et al., 2016; Valenciano et al., 2016; Goldstein et al., 2017; Ma et al., 2017; Monamele et al., 2017; Suntronwong et al., 2017; Korsun et al., 2018). More importantly, the non-subtyped H3N2 strains using the Luminex RVP assay in this study were further subjected to genetic sequencing, and the occurrence of mutations and reassortment was verified (unpublished data, not shown). However, we could not exclude the possibility that a portion of these 15 specimens were positive for both H1N1 and H3N2, and the H1N1 virus could not be detected by both assays because it has been reported that post-pandemic 2009 influenza H1N1 viruses have been antigenically drifting (Clark et al., 2017). More sensitive but complicated and time-consuming detection methods such as metagenomic analysis could be carried out for further verification.

In this study, we have randomly selected 96 clinical pneumonia specimens with negative results for all viruses by using the Luminex RVP assay and further tested these specimens by using the multiplex RT-PCR-electrochemical DNA sensor. However, no more influenza A-positive specimens were detected. It appears that the M gene is relatively conserved and that the primers/probes used for identifying influenza A viruses in both assays are credible.

According to the sequencing confirmation of the positive specimens, we report the disappearance of seasonal H1N1 (which was oseltamivir-resistant), demonstrating that there is no current clinical need to offer information regarding seasonal H1N1. However, the subtype information is helpful for our epidemiology and infection control colleagues and may be beneficial for antiviral susceptibility development.

In this study, four H7N9-positive specimens detected with the real-time PCR assay showed negative results using the multiplex RT-PCR-electrochemical DNA sensor. Compared to the real-time PCR results, the copy numbers of the four negative specimens detected using the multiplex RT-PCR-electrochemical DNA sensor were less than 10^2^ copies/mL, while the other 23 H7N9-positive specimens with viral copies greater than 10^2^ copies/mL showed positive results using the multiplex RT-PCR-electrochemical DNA sensor. Thus, although the sensitivity of the multiplex RT-PCR-electrochemical DNA has been found comparable to the real-time PCR regarding the detection of H5N1, H5N6, H7N9, H9N2, and H10N8, it was less sensitive in detection of H7N9 influenza virus. However, the advantage of the multiplex RT-PCR-electrochemical DNA sensor is that it can simultaneously detect and subtype influenza A viruses, whereas the focus of the real-time PCR kit is only one subtype; therefore, more time and more specimens will be consumed. We are currently designing a new batch of primers and probes for the H7N9 virus and aim to find one group with more sensitivity without cross-reaction with the other subtypes to further optimize the multiplex RT-PCR-electrochemical DNA sensor.

## Conclusion

A sensitive, reliable, simultaneous detection system generated based on multiplex asymmetric PCR coupled to an electrochemical DNA sensor was developed to simultaneously detect and subtype influenza A viruses. This system might serve as a better alternative for hospitals and clinical laboratories that must identify viral pathogens earlier in the treatment continuum.

## Author Contributions

LX performed the data analysis and drafted the manuscript. YZ, YD, TH, ZH, and CL performed the laboratory experiments. ZX and XJ participated in the study design and coordinated the drafting of the manuscript. All the authors read and approved the final manuscript.

## Conflict of Interest Statement

The authors declare that the research was conducted in the absence of any commercial or financial relationships that could be construed as a potential conflict of interest.
